# Exploring characteristics of COPD patients with clinical improvement after integrated disease management or usual care: post-hoc analysis of the RECODE study

**DOI:** 10.1186/s12890-020-01213-8

**Published:** 2020-06-18

**Authors:** Eline Meijer, Annelies E. van Eeden, Annemarije L. Kruis, Melinde R. S. Boland, Willem J. J. Assendelft, Apostolos Tsiachristas, Maureen P. M. H. Rutten-van Mölken, Marise J. Kasteleyn, Niels H. Chavannes

**Affiliations:** 1 Department of Public Health and Primary Care, Leiden University Medical Centre, PO Box 9600, 2300 RC Leiden, Netherlands; 2 Institute of Health, Policy & Management, Erasmus University Rotterdam, PO Box 1738, 3000 DR Rotterdam, Netherlands; 3 Department of Primary and Community Care, Radboud University Medical Centre, 6500 HB Nijmegen, Netherlands; 4 Health Economics Research Centre, Nuffield Department of Population Health, University of Oxford, Oxford, OX3 7LF UK; 5 Department of Pulmonology, Leiden University Medical Centre, Leiden, PO Box 9600, 2300 RC Leiden, Netherlands

**Keywords:** COPD, Disease management, Primary care, Integrated care, Quality of life

## Abstract

**Background:**

The cluster randomized controlled trial on (cost-)effectiveness of integrated chronic obstructive pulmonary disease (COPD) management in primary care (RECODE) showed that integrated disease management (IDM) in primary care had no effect on quality of life (QOL) in COPD patients compared with usual care (guideline-supported non-programmatic care). It is possible that only a subset of COPD patients in primary care benefit from IDM. We therefore examined which patients benefit from IDM, and whether patient characteristics predict clinical improvement over time.

**Method:**

Post-hoc analyses of the RECODE trial among 1086 COPD patients. Logistic regression analyses were performed with baseline characteristics as predictors to examine determinants of improvement in QOL, defined as a minimal decline in Clinical COPD Questionnaire (CCQ) of 0.4 points after 12 and 24 months of IDM. We also performed moderation analyses to examine whether predictors of clinical improvement differed between IDM and usual care.

**Results:**

Regardless of treatment type, more severe dyspnea (MRC) was the most important predictor of clinically improved QOL at 12 and 24 months, suggesting that these patients have most room for improvement. Clinical improvement with IDM was associated with female gender (12-months) and being younger (24-months), and improvement with usual care was associated with having a depression (24-months).

**Conclusions:**

More severe dyspnea is a key predictor of improved QOL in COPD patients over time. More research is needed to replicate patient characteristics associated with clinical improvement with IDM, such that IDM programs can be offered to patients that benefit the most, and can potentially be adjusted to meet the needs of other patient groups as well.

**Trial registration:**

Netherlands Trial Register, NTR2268. Registered 31 March 2010.

## Background

Chronic obstructive pulmonary disease (COPD) is a common respiratory disorder with a high burden of disease. It is a growing problem worldwide, due to continued exposure to risk factors such as smoking [1]. COPD treatment aims to improve quality of life (QOL) and daily functioning, and support smoking cessation [2]. Integrated disease management (IDM) programs consisting of coherent, multidisciplinary interventions were developed for COPD. A Cochrane review showed that COPD patients who participated in IDM programs had improved QOL and exercise tolerance, and fewer and shorter hospital admissions related to exacerbations. Although no effects were found on mortality, these findings seem promising [3]. Later IDM studies however showed mixed results on clinical outcomes [4–12]. For example, the pragmatic randomized clinical trial on effectiveness of integrated COPD management in primary care (RECODE) study, which had broad inclusion criteria, showed that IDM had no beneficial effect on QOL in COPD patients compared to usual care [13–15]. This study also showed that IDM was more costly and less cost-effective than usual care [14]. It is possible that IDM compares less favourably to the usual care control group when usual care is of higher quality [9]. Alternatively, it could be that only a subset of all COPD patients in primary care show clinical improvement from IDM [15]. In line with this, in the RECODE study IDM was more cost-effective in patients under 65 years than in older patients, but no differences in cost-effectiveness of IDM were found based on gender, dyspnea, lung function and socioeconomic status [14]. It has also been suggested that IDM may be more effective if unmotivated patients are excluded [16]. If the profile of COPD patients who show clinically relevant improvement with IDM can be elucidated, IDM programs can be used in this particular group of patients to maximize benefit. Similarly, it is important to know which patients benefit most from usual care for COPD, and which patients may need additional, or different type of, care.

As far as we know, no studies to date have examined this question in IDM programmes, although there are studies which addressed this in the context of self-management. These are relevant for the current investigation because self-management is an important element of IDM programs for COPD patients [3]. An individual patient data meta-analysis showed that self-management was more beneficial for COPD patients who were male, had more severe airflow limitations, had moderate self-efficacy scores (vs. low or high), or were obese [17]. These effects were only found for respiratory-related and all-cause hospitalizations, but no differences were found for health-related QOL, hospital days and mortality. It has also been shown that patients who are younger and live with a partner are more successful in self-management [18]. In addition, younger patients, patients who have more severe airflow obstruction, cardiac co-morbidity and have had influenza vaccination are more likely to adhere to an exacerbation action plan [19]. Furthermore, several disease and psychological characteristics (e.g., hyperinflation, dyspnoea, previous exacerbations, QOL, self-efficacy) are related to physical activity in COPD patients [20]. As such, it is likely that response to IDM also differs based on patient characteristics. The objective of the current study was to explore baseline patient characteristics that are independently related to clinical improvement with IDM or usual care (i.e. guideline-supported non-programmatic care). Independent relations are important as patients characteristics may be interrelated. For example, it has been shown that women with COPD have higher levels of depression compared to men, and that depressive symptoms are associated with increased perception of dyspnea [21–23]. Moreover, dyspnea in COPD patients is related to comorbidity [24]. We examined the following research questions (RQs):

1. Which baseline characteristics are related to clinically relevant improvement in COPD-specific health-related QOL after IDM or usual care, at 12-month and 24-month follow-up?
2. Does treatment condition (i.e. IDM or usual care) moderate relations between baseline characteristics and clinically relevant improvement?

## Methods

### Design

This exploratory post-hoc study is embedded within the RECODE study. The RECODE study was a pragmatic 1:1 cluster randomized controlled trial with 12-month and 24-month follow-up that assessed the long term (cost-)effectiveness of IDM incorporated in primary care in COPD patients [13, 15]. The primary outcome was the difference in health-related QOL measured by the Clinical COPD Questionnaire (CCQ), after 12 months of follow-up (regardless of clinically significant improvement). To assess whether effects were maintained the total study duration was 24 months. Forty clusters of primary care teams were randomized either to the IDM intervention or to usual care.

The IDM intervention included a two-day training in essential components of COPD IDM for general practitioners, practice nurses and specialised physiotherapists. They received information on proper diagnosis, optimizing medication adherence, motivational interviewing, smoking cessation counselling, applying self-management plans including early recognition and treatment of exacerbations, and physical (re)activation and nutritional support, and they were trained in using an IT application that aimed to facilitate communication within the team and with patients. Subsequently, each primary care team designed its own practice plan to implement IDM in daily practice. Usual care was based on Dutch general practice COPD guidelines, in line with GOLD guidelines [2, 25], and typically consisted of regular scheduled visits with a practice nurse with a main focus on spirometry or ad-hoc visits to a general practitioner in case of an exacerbation. IDM with different intervention components delivered by a multidisciplinary team was not regularly implemented in primary care. Practice nurses in the usual care group received a course on technical performance of spirometry only, to divert attention from topics related to our intervention. The study was conducted in The Netherlands between 2010 and 2013. Full details of the design, IDM intervention and usual care are provided elsewhere [13, 15].

### Participants

Patients were included if they were diagnosed according to national/international guidelines with COPD by their treating physician. Patients were invited for formal lung function assessment if spirometry data were unavailable. Exclusion criteria were terminal illness, dementia or cognitive impairment, abuse of hard drugs or alcohol and inability to fill in Dutch questionnaires. In total 1086 COPD patients were included. Participant characteristics are provided elsewhere [13, 14].

### Procedure

#### Measures

##### Predictor variables (baseline)

Several variables were assessed in the RECODE trial, of which those relevant to the current analyses are reported below.

***Socio-demographic factors.*** Participants provided their gender; age; living condition (alone vs. together); and socioeconomic status based on their educational level (recoded into ‘low education’ referring to no education or lower vocational education, or ‘no low education); and employment status.

***Lung function and symptoms.*** We extracted Forced Expiratory Volume in 1 s (FEV1), post-bronchodilator, % predicted according to age and height from medical files. FEV1 was measured by practice nurses or respiratory nurses if unknown. Dyspnea was assessed with the Medical Research Council [MRC] Dyspnea Scale), with 2 as cut-off value for dyspnea [26]. Data on the total number of moderate/severe exacerbations in the previous year were extracted from medical records, with an exacerbation being defined as a worsening of daily symptoms requiring oral prednisone and/or antibiotic courses and/or hospitalizations.

***Comorbidity.*** Comorbidity variables included presence of major cardiovascular disease, hypertension, diabetes and depression (yes/no). In addition, the Charlson co-morbidity index was used to assess overall comorbidity severity, with higher scores indicating more severe conditions and higher mortality risk according to comorbidities [27].

***Lifestyle, illness behaviour and knowledge*** We assessed smoking status (self-report); physical activity in Metabolic Equivalent Time [MET] minutes, measured by the International Physical Activity Questionnaire [IPAQ] [28]; and self-management, measured as taking initiative, investment behavior and level of self-efficacy with the Self-Management Scale-30 [SMAS-30] [29].

##### Outcome variables (12-month and 24-month follow-up)

***Clinical COPD Questionnaire (CCQ).*** Clinical improvement was assessed with the CCQ, a 10-item COPD-specific QOL questionnaire that includes a symptoms, functional and mental domain [30]. Each item is scored on a scale from 0 (best possible score) to 6 (worst possible score). The minimal clinically important difference (MCID) is a decline of 0.4 points [30, 31]. The CCQ was found to be responsive to change in previous studies [30, 32]. In this study two categorical variables were created in order to classify each participant as improved patient’ (i.e. decline in CCQ ≥ 0.4 between baseline and follow-up) or ‘unimproved patient’ (i.e. decline in CCQ < 0.4), at 12-month and 24-month follow-up, respectively. In addition, the numeric CCQ difference scores were used in sensitivity analyses (see Statistical analyses).

### Statistical analyses

Preliminary analyses were performed using descriptive statistics to examine how many patients showed clinically relevant improvement. We then performed four sets of logistic regression analyses to examine which baseline characteristics were associated with clinical improvement in the intervention and control group at 12-month and 24-month follow-up (RQ1). Specifically, for each time point and group we first performed univariable logistic regression analyses with the dichotomous CCQ improvement variable as outcome. Significant predictors (*p* < 0.05) were then added to the multivariable model to examine which characteristics were independently related to clinical improvement. Sensitivity analyses were performed by repeating this procedure with the numeric CCQ difference scores as outcome variables, using univariable and multivariable linear regression analyses. We also performed generalized linear mixed model (GLMM) analyses to account for the effect of clustering, with cluster team added as random effect (see Supplementary material). However, these models often failed to converge, possibly due to small cluster effects. The cluster covariance values were indeed very small and results of GLMM were very similar to logistic regression results.

For RQ2 we performed a set of hierarchical logistic regression analyses to examine whether predictors of clinical improvement differed significantly between the intervention and control group, i.e. moderation analysis. Specifically, we entered the main effects of predictors that were significant in the univariable analyses (in either group), as well as the treatment condition variable, in Step 1. The interaction between the treatment condition variable and a specific predictor (focusing on predictors that showed different effects in the multivariable analyses in the intervention and control group) was entered in Step 2. Participants with full data for the variables included in a specific model were included in the respective analysis. We ensured that assumptions of all analyses were met. Statistical analyses were performed using IBM Statistics SPSS version 23.

## Results

### Preliminary analyses

One hundred and eighteen (23%) patients in the intervention group of 514 patients that completed 12-month follow-up were classified as improved patients, compared to 134 (28%) in the control group of 476 patients. Furthermore, at 12-month follow-up 136 (26%) and 146 (31%) patients in the intervention and control group, respectively, showed a clinically significant deterioration, and 260 (51%) and 196 (41%) intervention and control patients, respectively, did not show clinically significant change. At 24-month follow-up, 57 patients in the intervention group (14% of 394 patients) and 55 patients in the control group (15% of 363 patients) were classified as improved patients. In addition, 195 (49%) intervention and 172 (47%) control group patients deteriorated compared to baseline, and 142 (36%) and 136 (37%), respectively did not show clinically significant change.

### Predictors of clinical improvement (RQ1)

#### Intervention group

##### Main analyses

In the univariable analyses, clinical improvement with IDM at 12-month follow-up was more likely in female and lower-SES patients, and in patients with an MRC dyspnea score > 2, and higher scores on the Charlson co-morbidity index (see Table 1). When these factors were combined in a multivariable analysis, the only independent predictors of clinical improvement with IDM were being female and having a MRC dyspnea score > 2. For 24-month follow-up, the univariable analyses showed that improved patients at 24 months were more likely to be younger and have a MRC dyspnea score > 2 (see Table 2). These effects remained significant in the multivariable model with these two variables as predictors of improvement.

**Table 1:** Predictors of clinical improvement with IDM in the intervention group (12-months follow-up): Logistic regression analyses

		Univariable analyses (*N* = 462–514)		Multivariable analysis (*N* = 461)	
Predictor	Value	Odds ratio (95% CI)	*p*-value	Odds ratio (95% CI)	*p*-value
***Socio-demographic factors***					
Gender	Female vs Male	1.84 (1.21–2.80)	< 0.01	1.75 (1.11–2.77)	0.02
Age	Each year	0.99 (0.98–1.01)	0.45		
Living alone	Yes vs No	1.35 (0.87–2.08)	0.18		
Low education	Yes vs No	1.78 (1.15–2.76)	0.01	1.57 (1.00–2.46)	0.051
Employment	Yes vs No	0.63 (0.38–1.04)	0.07		
***Lung function and symptoms***					
FEV1% predicted	Each % predicted	0.99 (0.98–1.00)	0.23		
Dyspnea - MRC score > 2	Yes vs No	2.15 (1.41–3.27)	< 0.001	1.79 (1.12–2.86)	0.01
Exacerbation frequency of previous year	Each exacerbation	1.07 (0.85–1.35)	0.59		
***Co-morbidity***					
Major cardiovascular disease	Yes vs No	1.20 (0.66–2.18)	0.55		
Hypertension	Yes vs No	1.07 (0.69–1.67)	0.75		
Diabetes	Yes vs No	1.12 (0.62–2.03)	0.71		
Depression	Yes vs No	1.67 (0.87–3.21)	0.12		
Charlson co-morbidity index	Each point	1.20 (1.02–1.41)	0.03	1.12 (0.94–1.33)	0.22
***Lifestyle, illness behaviour and knowledge***					
Current smoker	Yes vs No	1.07 (0.68–1.68)	0.77		
Physical activity (in MET)	Each minute	1.00 (1.00–1.00)	0.39		
Self-management	Taking initiatives - each point	0.99 (0.98–1.00)	0.12		
	Investment behavior - each point	0.99 (0.99–1.01)	0.78		
	Self-efficacy - each point	1.00 (0.99–1.01)	0.74		

Values are presented as odds ratios (95% confidence interval [CI]). Odds ratio > 1 indicates a greater likelihood of clinical improvement with IDM

Multivariable model Cox & Snell *R*^2^ = 0.05, Nagelkerke *R*^2^ = 0.08

*IDM* Integrated Disease Management, *FEV1* Forced Expiratory Volume in 1 s, post-bronchodilator, predicted according to age and height, *MRC* Medical Research Council Dyspnea Scale, *MET* Metabolic Equivalent Time

**Table 2:** Predictors of clinical improvement with IDM in the intervention group (24-months follow-up): Logistic regression analyses

		Univariable analyses (*N* = 359–394)		Multivariable analysis (*N* = 394)	
Predictor	Value	Odds ratio (95% CI)	*p*-value	Odds ratio (95% CI)	*p*-value
***Socio-demographic factors***					
Gender	Female vs Male	1.43 (0.81–2.52)	0.21		
Age	Each year	0.95 (0.93–0.98)	< 0.001	0.95 (0.92–0.97)	< 0.001
Living alone	Yes vs No	0.94 (0.51–1.74)	0.85		
Low education	Yes vs No	1.40 (0.77–2.54)	0.27		
Employment	Yes vs No	1.04 (0.54–1.98)	0.91		
***Lung function and symptoms***					
FEV1% predicted	Each % predicted	1.01 (1.00–1.02)	0.19		
Dyspnea - MRC score > 2	Yes vs No	1.85 (1.05–3.28)	0.03	2.28 (1.26–4.12)	0.01
Exacerbation frequency of previous year	Each exacerbation	1.12 (0.84–1.49)	0.45		
***Co-morbidity***					
Major cardiovascular disease	Yes vs No	1.43 (0.67–3.05)	0.35		
Hypertension	Yes vs No	0.66 (0.35–1.25)	0.20		
Diabetes	Yes vs No	1.19 (0.52–2.70)	0.68		
Depression	Yes vs No	1.42 (0.56–3.63)	0.47		
Charlson co-morbidity index	Each point	1.10 (0.88–1.37)	0.42		
***Lifestyle, illness behaviour and knowledge***					
Current smoker	Yes vs No	1.21 (0.66–2.21)	0.53		
Physical activity (in MET)	Each minute	1.00 (1.00–1.00)	0.37		
Self-management	Taking initiatives - each point	0.99 (0.97–1.01)	0.19		
	Investment behavior - each point	0.99 (0.97–1.01)	0.20		
	Self-efficacy - each point	0.99 (0.97–1.01)	0.25		

Values are presented as odds ratios (95% confidence interval [CI]). Odds ratio > 1 indicates a greater likelihood of clinical improvement with IDM

Multivariable model Cox & Snell *R*^2^ = 0.05, Nagelkerke *R*^2^ = 0.09

*IDM* Integrated Disease Management, *FEV1* Forced Expiratory Volume in 1 s, post-bronchodilator, predicted according to age and height, *MRC* Medical Research Council Dyspnea Scale, *MET* Metabolic Equivalent Time

##### Sensitivity analyses

Univariable linear regression analyses showed that only lower SES predicted change in CCQ at 12 months (i.e., the numeric CCQ difference variable), such that those with lower SES showed more improvement from baseline to 12-month follow-up than those with middle or higher SES (*b* = − 0.17, 95% CI = − 0.29;-0.04, *p* = 0.01). Improvement in CCQ from baseline to 24-month-follow-up was related to being female, younger, and having a depression in univariable analyses. In the multivariable model, the effects of gender (*b* = − 0.15, 95% CI = − 0.30;0.00, *p* = 0.04) and age (*b* = 0.01, 95% CI = 0.00;0.02, *p* = 0.01) remained significant, whereas the effect of depression became nonsignificant (*b* = − 0.25, 95% CI = − 0.51;0.02, *p* = 0.07). MRC dyspnea score did not emerge as a significant predictor in these analyses.

#### Control group

##### Main analyses

Univariable logistic regression analyses in the control group showed that only MRC dyspnea score > 2 was significantly associated with clinical improvement at 12-month follow-up (see Table 3). We therefore did not perform multivariable analyses. For 24-month follow-up, univariable logistic regression analyses showed different predictors of clinical improvement at 24-month follow-up, which was more likely for patients with major cardiovascular disease, hypertension or depression, and for patients with a higher Charlson co-morbidity index (see Table 4). Only depression remained significant in the multivariable model.

**Table 3:** Predictors of clinical improvement with IDM in the control group (12-months follow-up): Logistic regression analyses

		Univariable analyses (*N* = 443–476)	
Predictor	Value	Odds ratio (95% CI)	*p*-value
***Socio-demographic factors***			
Gender	Female vs Male	1.10 (0.74–1.65)	0.64
Age	Each year	0.99 (0.97–1.01)	0.16
Living alone	Yes vs No	1.00 (0.66–1.53)	0.99
Low education	Yes vs No	1.24 (0.82–1.88)	0.32
Employment	Yes vs No	1.44 (0.93–2.24)	0.11
***Lung function and symptoms***			
FEV1% predicted	Each % predicted	1.00 (0.99–1.01)	0.96
Dyspnea - MRC score > 2	Yes vs No	1.85 (1.21–2.82)	< 0.01
Exacerbation frequency of previous year	Each exacerbation	1.17 (0.94–1.46)	0.17
***Co-morbidity***			
Major cardiovascular disease	Yes vs No	1.22 (0.73–2.03)	0.45
Hypertension	Yes vs No	1.19 (0.79–1.80)	0.40
Diabetes	Yes vs No	1.32 (0.77–2.29)	0.32
Depression	Yes vs No	1.18 (0.62–2.27)	0.61
Charlson co-morbidity index	Each point	1.11 (0.95–1.29)	0.20
***Lifestyle, illness behaviour and knowledge***			
Current smoker	Yes vs No	0.97 (0.64–1.47)	0.88
Physical activity (in MET)	Each minute	1.00 (1.00–1.00)	0.96
Self-management	Taking initiatives - each point	0.99 (0.98–1.01)	0.32
	Investment behavior - each point	1.00 (0.99–1.01)	0.81
	Self-efficacy - each point	0.99 (0.98–1.00)	0.19

Values are presented as odds ratios (95% confidence interval [CI]). Odds ratio > 1 indicates a greater likelihood of clinical improvement with IDM

*IDM* Integrated Disease Management, *FEV1* Forced Expiratory Volume in 1 s, post-bronchodilator, predicted according to age and height, *MRC* Medical Research Council Dyspnea Scale, *MET* Metabolic Equivalent Time

**Table 4:** Predictors of clinical improvement with IDM in the control group (24-months follow-up): Logistic regression analyses

		Univariable analyses (*N* = 343–363)		Multivariable analysis (*N* = 354)	
Predictor	Value	Odds ratio (95% CI)	*p*-value	Odds ratio (95% CI)	*p*-value
***Socio-demographic factors***					
Gender	Female vs Male	1.70 (0.96–3.03)	0.07		
Age	Each year	0.98 (0.95–1.00)	0.10		
Living alone	Yes vs No	0.82 (0.44–1.52)	0.53		
Low education	Yes vs No	0.99 (0.54–1.81)	0.98		
Employment	Yes vs No	0.99 (0.53–1.88)	0.99		
***Lung function and symptoms***					
FEV1% predicted	Each % predicted	1.01 (1.00–1.03)	0.17		
Dyspnea - MRC score > 2	Yes vs No	1.54 (0.84–2.82)	0.16		
Exacerbation frequency of previous year	Each exacerbation	1.24 (0.94–1.65)	0.13		
***Co-morbidity***					
Major cardiovascular disease	Yes vs No	2.12 (1.08–4.15)	0.03	1.86 (0.81–4.26)	0.14
Hypertension	Yes vs No	2.16 (1.21–3.86)	0.01	2.00 (0.98–4.08)	0.06
Diabetes	Yes vs No	1.49 (0.71–3.11)	0.29		
Depression	Yes vs No	2.74 (1.26–5.96)	0.01	2.60 (1.06–6.38)	0.04
Charlson co-morbidity index	Each point	1.42 (1.14–1.75)	0.001	1.07 (0.77–1.47)	0.70
***Lifestyle, illness behaviour and knowledge***					
Current smoker	Yes vs No	1.05 (0.58–1.91)	0.88		
Physical activity (in MET)	Each minute	1.00 (1.00–1.00)	0.52		
Self-management	Taking initiatives - each point	1.00 (0.98–1.02)	0.98		
	Investment behavior - each point	1.01 (0.99–1.02)	0.62		
	Self-efficacy - each point	0.99 (0.97–1.01)	0.33		

Values are presented as odds ratios (95% confidence interval [CI]). Odds ratio > 1 indicates a greater likelihood of clinical improvement with IDM

Multivariable model Cox & Snell *R*^2^ = 0.05, Nagelkerke *R*^2^ = 0.08

*IDM* Integrated Disease Management, *FEV1* Forced Expiratory Volume in 1 s, post-bronchodilator, predicted according to age and height, *MRC* Medical Research Council Dyspnea Scale, *MET* Metabolic Equivalent Time

##### Sensitivity analyses

Univariable linear regression analyses showed that patients with higher FEV1% predicted scores showed stronger improvement in CCQ from baseline to 12-month follow-up (*b* = − 0.004, 95% CI = − 0.01;0.00, *p* = 0.01). Improvement at 24 months was related to higher FEV1% predicted scores, and presence of hypertension and depression in the univariable analyses. The multivariable model showed that improvement was significantly related to higher FEV1 scores (*b* = − 0.01, 95% CI = − 0.01;0.00, *p* = 0.04) and hypertension (*b* = − 0.18, 95% CI = − 0.34;-0.02, *p* = 0.03), but not to depression (*b* = − 0.25, 95% CI = − 0.51;0.02, *p* = 0.07).

### Moderation by treatment condition (RQ2)

The analyses above showed that clinically relevant improvement in CCQ scores at 12-month follow-up was independently related to gender and MRC in the IDM intervention group, but the effect of gender was not found in the usual care control group. Furthermore, clinically relevant improvement at 24-months was independently related to MRC > 2 and younger age in the intervention group, whereas the multivariable analyses for the control group only showed an independent effect of depression. Follow-up moderation analyses showed no significant interactions between treatment condition and gender in predicting improvement at 12-months, nor between treatment condition and age, MRC > 2 or depression, respectively, in predicting improvement at 24-months (see Table 5).

**Table 5:** Moderation of prediction of clinically relevant improvement in CCQ score by condition: Logistic regression analyses

			12-month follow-up (*N* = 902)		24-month follow-up (*N* = 728)	
Model	Predictor	Value	Odds ratio (95% CI)	*p*-value	Odds ratio (95% CI)	*p*-value
Step 1	Gender	Female vs Male	1.28 (0.94–1.74)	0.12		
	Age	Each year			0.96 (0.94–0.98)	< 0.001
	Low education	Yes vs No	1.34 (0.98–1.82)	0.06		
	Dyspnea - MRC score > 2	Yes vs No	1.84 (1.33–2.53)	< 0.001	1.61 (1.03–2.51)	0.04
	Major cardiovascular disease	Yes vs No			1.63 (0.86–3.08)	0.13
	Hypertension	Yes vs No			1.00 (0.60–1.66)	1.00
	Depression	Yes vs No			1.33 (0.67–2.66)	0.42
	Charlson co-morbidity index	Each point	1.10 (0.98–1.24)	0.12	1.12 (0.89–1.41)	0.34
	Condition	Intervention vs Control	0.72 (0.53–0.98)	0.04	0.90 (0.59–1.36)	0.61
Step 2 (12-month)	Condition*Gender		1.85 (1.00–3.44)	0.05		
Step 2A (24-month)	Condition*Age				0.98 (0.94–1.02)	0.25
Step 2B (24-month)	Condition*Dyspnea - MRC score > 2				1.08 (0.46–2.57)	0.86
Step 2C (24-month)	Condition*Depression				0.56 (0.16–1.97)	0.37

Step 1 included predictor variables that were significant in the multivariable analysis in the intervention and/or control group, as well as the condition variable. Interactions terms were added in Step 2 in separate models. Values are presented as odds ratios (95% confidence interval [CI])

*MRC* Medical Research Council Dyspnea Scale

## Discussion

This exploratory study examined which COPD patients benefit most from an integrated disease management (IDM) program or usual care in a primary care setting, in terms of clinically relevant improvement in COPD specific health-related QOL (CCQ), in a study that revealed overall no effect. Baseline MRC dyspnea scores appeared most important for clinical improvement (i.e. clinically significant improvement in CCQ), such that patients with more severe dyspnea in either group were more likely to have improved at 12-month follow-up, and this effect remained at 24-month follow-up in the intervention group. This finding is in line with previous studies showing that MRC dyspnea scores are important for clinical improvement [33, 34]. In line with previous findings from the RECODE study, MRC did not emerge as a predictor of numeric CCQ difference scores (regardless of clinically significant improvement) in the sensitivity analyses. Arguably, clinically significant improvement in CCQ scores likely is more important for patients than (mere) statistically significant improvement. Furthermore, and as can be expected, it suggests that patients who were worse off at the start of an intervention had more room for improvement. In line with the latter, control group patients who were more depressed at baseline were more likely to have improved CCQ scores at 24-months follow-up. We furthermore found that clinical improvement in QOL at 24-month follow-up was more likely in younger patients in the IDM group. This finding relates to results of the cost-effectiveness study, showing that IDM is more cost-effective among patients aged under 65 [14]. Finally, female patients benefitted more from IDM than male patients at 12-month (but not 24-month) follow-up. This finding contrasts a previous meta-analysis on self-management, which showed that male COPD patients benefitted more than female patients [17]. In the current study, female patients reported higher baseline CCQ scores than male patients (*M*_female_ = 1.61, *SD*_female_ = 1.02; *M*_male_ = 1.41, *SD*_male_ = 0.92, *p* = 0.001) indicating higher disease burden, such that they may have had more room for improvement in general. In line with this reasoning, female patients in previous studies showed more emotional and psychosocial benefit from pulmonary rehabilitation compared to males [35, 36]. Furthermore, for the same degree of airflow limitation females report more dyspnea, more limitations in physical activity and worse QOL scores in comparison to males [21, 36–39]. However, the effect of gender found in the current study was independent of dyspnea scores.

Finally, although some predictors were related to improved QOL regardless of how improvement was defined (i.e. dichotomized minimal clinical difference of 0.40 points reduction vs. numeric difference scores), FEV1% predicted scores only predicted numeric change scores but not clinically relevant improvement. This could be because both (small) changes in CCQ scores and FEV1% predicted scores are not directly noticeable to patients in daily life. That is, patients’ objective lung function may not be directly related to their experienced level of disease burden, such that patients with relatively well maintained lung function may experience high burden of disease, whereas patients with a worse lung function may experience fewer limitations [40, 41]. Furthermore, patients with a lower burden of disease often do not feel the need to participate in a pulmonary rehabilitation programme [42].

## Limitations

This study has several limitations. First, this post-hoc study was embedded in the RECODE study which had a different primary aim and was not powered for subgroup analyses, and several statistical tests were performed (although the multivariable models reduce the risk of type-1 error resulting from multiple testing). The results of our study should therefore be considered as hypothesis generating, rather than confirming or rejecting specific directional hypotheses with regard to treatment type and patient subgroups [43]. Second, it is not clear whether clinical improvement could be attributed to IDM or care as usual, or was caused by factors other than treatment type. That is, an evaluation study showed wide variety in the implementation of the RECODE program across the primary care teams, but we could not take the extent of IDM implementation into consideration [16]. Furthermore, the control group of the RECODE study received ‘usual care’, but this care was affected by national reforms in COPD care during the study period and included aspects of IDM as well [15]. Third, it is possible that regression to the mean played a role in improved CCQ scores. However, a substantial group of patients in this study did not improve or even deteriorated. Importantly, we assessed which patient characteristics were related to improvement and not merely whether patients improved. Fourth, although we included a range of patient characteristics, it is possible that we missed relevant patient variables that may moderate effects, such as obesity [17].

## Conclusions

In this study, more severe dyspnea was the most important predictor of clinically improved QOL, regardless of treatment type. This suggests that these patients have most room for improvement, but should not necessarily be assigned to an IDM program, because the interaction term was not statistically significant. Clinical improvement with IDM was associated with female gender (12-months) and being younger (24-months), and improvement with usual care was associated with having a depression (24-months). More research is needed to replicate patient characteristics associated with clinical improvement with IDM, such that IDM programs can be offered to patients that benefit the most, and can potentially be adjusted to meet the needs of other patient groups as well. In addition, it would be useful to know which patients respond best to which intervention components [44].

## Supplementary information

**Additional file 1.** Supplementary Material. Predictors of clinical improvement with IDM in the intervention group (12-months follow-up): Generalized linear mixed model.

## Data Availability

The datasets used and/or analyzed during the current study are available from the corresponding author on reasonable request.

## Abbreviations

CCQ: Clinical COPD Questionnaire
COPD: Chronic obstructive pulmonary disease
FEV1: Forced Expiratory Volume in 1 second
IDM: Integrated disease management
IPAQ: International Physical Activity Questionnaire
MCID: Minimal clinically important difference
MET: Metabolic Equivalent Time
MRC: Medical Research Counsil
RECODE: Acronym for “randomized clinical trial on effectiveness of integrated COPD management in primary care”
RQ: Research question
SMAS-30: Self-Management Scale-30
QOL: Quality of life
