# Supplementary material for: Exploring characteristics of COPD patients with clinical improvement after integrated disease management or usual care: post-hoc analysis of the RECODE study
Source: BMC Pulm Med. 2020 Jun 18;20:176. doi: 10.1186/s12890-020-01213-8 (PMC7302138; doi:10.1186/s12890-020-01213-8)
Supplement: Supplementary file 1 — Additional file 1. Supplementary Material. Predictors of clinical improvement with IDM in the intervention group (12-months follow-up): Generalized linear mixed model. [file 12890_2020_1213_MOESM1_ESM.docx]

| ***Supplementary Material.* Predictors of clinical improvement with IDM in the intervention group (12-months follow-up): Generalized linear mixed model** | | | | | | | |
| --- | --- | --- | --- | --- | --- | --- | --- |
|  | | |  | **Univariable analyses**  **(*N* = 462-514)** | | **Multivariable analysis**  **(*N* = 484)** | |
| **Predictor** | | | **Value** | **Odds ratio**  **(95% CI)** | **p-value** | **Odds ratio**  **(95% CI)** | **p-value** |
| ***Socio-demographic factors*** | | |  |  |  |  |  |
| Gender | | | Female vs Male | 1.79 (1.20-2.67) | < 0.01 | 1.68 (1.08-2.63) | 0.02 |
| Age | | | Each year | 0.99 (0.98-1.01) | 0.57 |  |  |
| Living alone | | | Yes vs No | 1.31 (0.85-2.02) | 0.22 |  |  |
| Low education | | | Yes vs No | 1.72 (0.99-2.99) | 0.06 |  |  |
| Employment | | | Yes vs No | 0.63 (0.38-1.05) | 0.08 |  |  |
| ***Lung function and symptoms*** | | |  |  |  |  |  |
| FEV1 % predicted | | | Each % predicted | 0.99 (0.98-1.00) | 0.24 |  |  |
| Dyspnea - MRC score >2 | | | Yes vs No | 2.11 (1.47-3.02) | < 0.001 | 1.79 (1.21-2.64) | < 0.01 |
| Exacerbation frequency of previous year | | | Each exacerbation | 1.07 (0.90-1.28) | 0.44 |  |  |
| ***Co-morbidity*** | | |  |  |  |  |  |
| Major cardiovascular disease | | | Yes vs No | 1.23 (0.73-2.06) | 0.44 |  |  |
| Hypertension | | | Yes vs No | 1.08 (0.62-1.86) | 0.79 |  |  |
| Diabetes | | | Yes vs No | 1.11 (0.69-1.79) | 0.66 |  |  |
| Depression | | | Yes vs No | 1.59 (1.00-2.53) | 0.048 | 1.21 (0.74-1.98) | 0.45 |
| Charlson co-morbidity index | | | Each point | 1.20 (1.05-1.38) | 0.01 | 1.12 (0.96-1.31) | 0.16 |
| ***Lifestyle, illness behaviour and knowledge*** | | |  |  |  |  |  |
| Current smoker | | Yes vs No | | 1.02 (0.74-1.40) | 0.93 |  |  |
| Physical activity (in MET) | | | Each minute | 1.00 (1.00-1.00) | 0.49 |  |  |
| Self-management | Taking initiatives - each point | | | 0.99 (0.98-1.00) | 0.06 |  |  |
|  | Investment behavior - each point | | | 0.99 (0.99-1.00) | 0.77 |  |  |
|  | Self-efficacy - each point | | | 0.99 (0.99-1.01) | 0.73 |  |  |
| Values are presented as odds ratios (95% confidence interval [CI]). Odds ratio > 1 indicates a greater likelihood of clinical improvement with IDM. IDM=Integrated Disease Management; FEV1=Forced Expiratory Volume in 1 second, post-bronchodilator, predicted according to age and height; MRC=Medical Research Council Dyspnea Scale; MET= Metabolic Equivalent Time. | | | | | | | |
